# The Impact of Genetic Mutations on the Efficacy of Immunotherapies in Lung Cancer

**DOI:** 10.3390/ijms252211954

**Published:** 2024-11-07

**Authors:** Ki Lui, Kwok-Kuen Cheung, Winnie Wing-Man Ng, Yanping Wang, Doreen W. H. Au, William C. Cho

**Affiliations:** 1 Department of Health Sciences, School of Nursing and Health Sciences, Hong Kong Metropolitan University, Hong Kong SAR, China; ypwang@hkmu.edu.hk (Y.W.); dau@hkmu.edu.hk (D.W.H.A.); 2 Department of Rehabilitation Sciences, The Hong Kong Polytechnic University, Hong Kong SAR, China; alexkk.cheung@polyu.edu.hk; 3 School of Nursing, The Hong Kong Polytechnic University, Hong Kong SAR, China; winnie.wm.ng@polyu.edu.hk; 4 Department of Clinical Oncology, Queen Elizabeth Hospital, Hong Kong SAR, China

**Keywords:** chemoresistance, genetic mutation, immunotherapy, lung cancer

## Abstract

Lung cancer is the leading cause of cancer-related mortality worldwide, primarily driven by genetic mutations. The most common genetic alterations implicated in lung cancer include mutations in *TP53*, *KRAS*, *KEAP1*, *NF1*, *EGFR*, *NRF2*, *ATM*, *ALK*, *Rb1*, *BRAF*, *MET*, and *ERBB2*. Targeted therapies have been developed to inhibit cancer growth by focusing on these specific genetic mutations. However, either the mutations are undruggable or the efficacy of these therapies is often compromised over time due to the emergence of drug resistance, which can occur through additional mutations in the targeted protein or alternative growth signaling pathways. In recent years, immunotherapy has emerged as a promising approach to enhance the effectiveness of cancer treatment by leveraging the body’s immune system. Notable advancements include immune checkpoint inhibitors, monoclonal antibodies targeting cell surface receptors, antibody–drug conjugates, and bispecific antibodies. This review provides an overview of the mechanisms of FDA-approved immunotherapeutic drugs, offering an updated perspective on the current state and future developments in lung cancer therapy. More importantly, the factors that positively and negatively impact the immunotherapy’s efficacy will also be discussed.

## 1. Introduction

The International Agency for Research on Cancer (IARC), a specialized cancer agency of the World Health Organization (WHO), reports that lung cancer is the most lethal and the leading cause of death among all cancers. In 2022 alone, it is estimated to cause approximately 1.8 million deaths, accounting for 18.7% of all cancer-related deaths, and results in 2.5 million new diagnoses, representing 12.4% of all cancer cases globally [1]. These distressing statistics indicate that our current understanding and medical intervention for lung cancer are inadequate, requiring further efforts in combating this disease. Furthermore, statistical projections suggest that the global cancer burden is set to double by 2050 [1]. Consequently, the need for comprehensive cancer research to develop effective therapy is more critical than ever.

Over several decades dedicated to cancer research, researchers have amassed a substantial body of knowledge regarding cancer initiation, progression, and metastasis. We are now in a new era when we urge to leverage cutting-edge technologies to combat cancer by specifically targeting these distorted pathways. This review aims to summarize the genetic mutations associated with lung cancer and discuss the genetic mutation biomarkers to predict the efficacy of immunotherapy. In recent years, immunotherapy—particularly PD-1/PD-L1 checkpoint inhibitors—has shown promise in treating a range of cancers. We will discuss recent advancements in immunotherapy in lung cancer therapy, covering topics such as PD-1/PD-L1 checkpoint inhibitors, monoclonal antibodies that target cell surface receptors, antibody–drug conjugates, and bispecific antibodies. Additionally, we will examine the factors that influence the effectiveness of immunotherapy, both positively and negatively.

## 2. Genetic Mutations in Lung Cancer and the Pathways to Drug Resistance

There are two major types of lung cancer: Non-Small Cell Lung Cancer (NSCLC) and Small Cell Lung Cancer (SCLC), accounting for 85% and 15% of total lung cancer cases, respectively [2]. SCLC is notably more aggressive, with an overall 5-year survival rate of only 3–7%, compared to 28% for NSCLC [3]. Additionally, over 95% of SCLC cases are linked to smoking [4]. In both types of lung cancer, somatic mutations contribute to the inhibition of growth suppressors, promotion of immune escape, and activation of various oncogenes, particularly epidermal growth factor receptor (EGFR) mutations in non-smokers [5].

The clonal evolution of cancer is a multifaceted process in which a cell must undergo and accumulate a series of genetic mutations sequentially to transform into a cancerous clone, which propels the formation of a cancerous mass [6]. Many critical gene mutations offer different growth and survival benefits to cancer cells, as systematically summarized by Hannahan; the 14 characteristic alterations in all cancers include enhancement in proliferative signals and genome instability, suppression in tumor suppressors, resistance to apoptosis, escape from immunity destruction, etc. [7]. These processes involved in cancer formation are well defined, and the pertinent inquiry is whether it is feasible to eradicate cancerous cells by rectifying the druggable mutation(s) in cancer or activating the immune responses to eliminate cancer. The answer is feasible but extremely difficult.

### 2.1. TP53 Mutations

Among all gene mutations in cancer, the tumor suppressor *p53 (TP53)* gene is the most commonly mutated gene in 50% of all cancers, while the TCGA Cancer Genome Atlas Program data show 65%, a higher *TP53* mutation rate in all lung cancers. The AACR GENIE project further showed a differential mutation rate in *TP53* in two types of lung cancers: about 46% of lung adenocarcinoma and 78% of squamous lung cancer acquired *TP53* mutations [8]. Among these lung cancer samples, various missense mutations in the DNA binding region have been detected, with higher prevalent hotspots on amino acids R175, R248, and R273 [8,9]. This agrees with the TCGA database shown in Table 1, which shows the prevalence of the top gene mutations and their mutation hotspots in lung cancer.

TP53 is a transcription factor, functioning as a guardian of the genome to trigger cell cycle arrest or cell death by sensing DNA damage [38,39] and oxidative stress [40] that could lead to gene mutations. Upon detection of these damages, TP53 is phosphorylated by various DNA damage response (DDR) kinases, such as ATM, ATR, DNA-PK, Chk1, and Chk2, to amino acids serine 15 (S15) and serine 20 (S20) along with other post-translational modifications by other modifying enzymes [41,42,43,44,45]. These modifications stabilize TP53 from degradation and facilitate the oligomerization transition from inactive monomers to active tetramers [46,47]. Subsequently, these tetramers bind to TP53-responsive genes, initiating gene transcription of cell cycle arrest and/or apoptosis regulatory proteins [48]. These *TP53* hotspot mutations mostly obstruct TP53’s ability to bind to DNA and sometimes interfere with its oligomerization, hindering the transcription of cell cycle arrest and/or apoptosis regulatory proteins. Thus, it provides a survival advantage to mutated cells that would typically be destined for death or arrest. Furthermore, cells with a heterozygous mutation in *TP53* express both wild-type and mutant TP53 proteins, in which mutant TP53 acts as a dominant negative mutant suppressing the wild-type TP53 functions on the other allele [49], inferring that a single allelic mutation is sufficient to block TP53 functions.

Even though 35–50% of cancers do not have any *TP53* mutation, it has been shown that various proteins can inhibit wild-type TP53 functions by accelerating its degradation [50,51,52,53,54,55,56,57] or blocking its oligomerization [51,58,59], such as Rab-like protein 1A (RBEL1A) [58]. Therefore, TP53 inhibition is viewed as a critical turning point and a major bottleneck in cancer development. Once TP53’s function is blocked, cancer can thrive.

Given that TP53 is often inhibited in cancer, it is logical to consider potential treatments that restore TP53 function. One such approach is utilizing oncolytic adenovirus gene therapy to deliver *TP53* into cancer, such as Gendicine [60] and Oncorine [61], which has been approved for treating cancer by the China Food and Drugs Administration (CFDA). However, this treatment has not been widely adopted in other countries, potentially due to various factors leading to unsatisfactory clinical results [62]. In addition to gene therapy, numerous pharmacologic small molecules [63], including Navtemadlin [64] and Idasanutlin [65], have been developed over the decades to restore TP53 functions or inhibit mutant TP53 functions. Despite these advancements, none have yet received approval from the U.S. Food and Drug Administration (FDA).

Mutations in *TP53* have been linked to resistance to various types of drugs, including but not limited to paclitaxel [66], cisplatin [67], and doxorubicin [68]. Different drugs trigger cancer cell death through unique upstream mechanisms, but ultimately, they all converge on the central death pathway involving TP53. Consequently, in the absence of TP53’s normal function, these drugs lose their effectiveness. Moreover, studies have shown that loss of TP53 function increases cancer cells’ resistance to immunotherapy by lowering antigen presentation in a lung cancer mouse model with high mutation burden and heterogeneity [69]. A poorer clinical outcome has also been observed in patients with *TP53* mutations with metastatic tumors treated by immunotherapy [70]. On the contrary, a study has shown that *TP53* and *K-Ras* co-mutations in lung adenocarcinoma correlate with the expression of PD-L1 on tumors for immune escape, and it has indicated substantial clinical benefits of PD-1 inhibitors in treating patients with these two genetic mutations [71]. More research is needed to confirm whether TP53 plays a critical role in immunotherapy efficacy.

### 2.2. K-Ras Mutations

*K-Ras* mutations represent the second most prevalent mutation, accounting for 16% of all mutations observed in lung cancer in the TCGA database. K-Ras, a growth-promoting GTPase and member of the Ras superfamily, is tightly regulated through a cycle of activation and deactivation, transitioning between the GTP-bound Ras (active) and GDP-bound Ras (inactive) state [15]. However, *K-Ras* mutations, especially on codon 12, such as G12C (the most prevalent form), G12V, and G12D, resist this regulation and maintain a perpetually active state that provides a continuous growth signal to cancer cells. Patients with these *K-Ras* mutations have poorer clinical outcomes and lower overall survival (OS) [72,73,74].

The previous consensus held that *Ras* mutations were undruggable due to their compact structure, consisting of only 189 amino acids. However, the market introduction of the K-Ras G12C inhibitor, Sotorasib, challenged this notion. The result of a small-scale (345 patients) CodeBreaK 200 Phase 3 Clinical Trial (NCT04303780) demonstrated that Sotorasib had a better progression-free survival (PFS) than docetaxel. During the median follow-up period of 17.7 months, the median PFS of Sotorasib treatment is 5.6 months (95% CI: 4.3–7.8 months), while the median PFS of docetaxel is 4.5 months (95% IC: 3.0–5.7 months) in NSCLC patients with a *K-Ras G12C* mutation [75]. In 2021, based on the positive results of the CodeBreaK 100 Clinical Trial (NCT03600883), Sotorasib received accelerated approval from the U.S. FDA to treat NSCLC. Mechanistically, Sotorasib covalently links to the mutant cysteine residue, which is only present on the G12C mutation site, thereby favoring the GDP-bound inactive state of mutant K-Ras and effectively switching off its growth signal [75,76,77]. Despite the initial excitement and anticipation for a clinical breakthrough with the K-Ras G12C inhibitor in cancer treatment, the FDA recently rejected the supplemental new drug application for Sotorasib. The Oncologic Advisory Drug Committee cited the reason that the submitted Phase 3 CodeBreaK 200 Clinical Trial (NCT04303780) data were interpreted unreliably and requested a Phase 4 post-marketing study to be completed before February 2028 [78,79]. So, additional data will be collected to ascertain whether Sotorasib will result in superior survival outcomes compared to other chemotherapy in patients with NSCLC.

Adagrasib is another K-Ras G12C inhibitor, a covalent coupler of the mutant cysteine residue, which has shown a comparable PFS, similar to Sotorasib, and the majority of patients experienced tolerable adverse side effects [80,81,82]. The KRYSTAL-1 Clinical Trial (NCT03785249) recruited 116 NSCLC patients who had *K-Ras G12C* mutation and had previously been treated with both chemotherapy and immunotherapy. At the median follow-up period of 12.9 months, the study found that the median PFS treated by Adagrasib was 6.5 months (95% CI: 4.7–8.4). At the median follow-up period of 15.6 months, the study found that the median OS was 12.6 months (95% CI: 9.2–19.2) [81,83]. Furthermore, the KRYSTAL-1 Clinical Trial has demonstrated that Adagrasib is effective in treating *K-Ras G12C-*mutated NSCLC with brain metastases. Radiographic evaluation of 19 patients revealed an intracranial clinical response rate of 42%, a disease control rate of 90%, a PFS of 5.4 months, and a median OS of 11.4 months over a median follow-up period of 13.7 months [84]. This evidence indicates that Adagrasib provides significant clinical benefits in treating both primary lung cancer and metastatic brain tumors. Following the successful demonstration of clinical benefits in the Phase 3 KRYSTAL-12 Clinical Trial (NCT04685135), the FDA has approved Adagrasib for the treatment of *K-Ras G12C*-mutated cancer. Currently, no other medication is available to target *K-Ras* mutants. A recent study has demonstrated that RGS3, a Ras GTPase-activating protein (GAP), can deactivate mutant K-Ras by promoting its transition to the GDP-bound inactive state [85]. This approach represents a potential novel strategy for targeting K-Ras in future therapeutic developments.

K-Ras also contributes to drug resistance. For instance, lung cancer cells with *EGFR* mutations that have metastasized to the brain can develop resistance to Osimertinib, a third-generation EGFR tyrosine kinase inhibitor (TKI), through the acquisition of an additional *K-Ras G12V* mutation [86]. These cancer cells can circumvent the EGFR-mediated growth signal blockade by developing an alternative growth pathway, such as a *K-Ras* mutation-driven growth signal. However, it is possible to resensitize these cancer cells to Osimertinib through a combined treatment with Trametinib, a MEK inhibitor, which blocks the downstream signals of K-Ras [86]. Furthermore, cancer cells resist the K-Ras G12C inhibitor by bypassing the growth signal from K-Ras through acquiring additional mutations in various pathways, such as gain of function of *ALK*, *BRAF*, and loss of function of *NF1* and *PTEN* mutations [87]. The common phenomenon that cancer can devise alternative strategies to circumvent drug treatment inhibition and engage in new growth pathways to flourish is a significant challenge. This suggests that we may need to adopt a different approach to cancer treatment, such as utilizing a combination of inhibitors to obstruct the majority of cancer growth pathways, thereby eliminating all potential avenues for cancer proliferation. However, this strategy may result in more severe patient side effects and may not necessarily yield beneficial outcomes.

The overexpression of K-Ras G12V mutants up-regulates PD-L1 in NSCLC to promote cancer’s immune escape via the epithelial-to-mesenchymal transition (EMT) [13]. The result suggests that NSCLC cells with a *K-Ras G12V* mutation are more susceptible to checkpoint inhibitors, suggesting that immunotherapy has significant potential in treating lung cancer with a K-Ras mutation.

### 2.3. Other Mutations

According to the TGCA database, about 14%, 11%, and 9% of lung cancer possess mutations in Kelch Like ECH Associated Protein 1 (*KEAP1*), Neurofibromatosis type 1 (*NF1*), and Epidermal growth factor receptor (*EGFR*), respectively. These are the third to fifth top mutations in lung cancer.

KEAP1 functions as an E3 ligase and is a critical regulator of the NRF2 transcription factor, essential for cellular protection against oxidative damage [88]. Under normal conditions, KEAP1 binds to NRF2, targeting it for ubiquitination and subsequent proteasomal degradation, thereby maintaining controlled NRF2 levels [89]. However, in cancer cells with mutated KEAP1, this regulatory mechanism is disrupted, resulting in the accumulation of NRF2. This accumulation activates a series of genes that protect cancer cells from oxidative stress, thereby promoting their survival and growth [17]. Additionally, gain-of-function mutations in *NRF2* are observed in 8% of lung cancer cases, according to the TGCA database. Patients with *KEAP1-NRF2* co-mutations in lung cancer exhibit poorer overall survival rates [90] and increased resistance to chemotherapy [91].

Recent studies demonstrate that KEAP1 downregulates PD-L1 expression in cancer cells via ubiquitination and proteasome-mediated degradation and that overexpression of KEAP1 enhances cytotoxic T cell activation in vivo [92]. Conversely, mutations in *KEAP1* are associated with a reduction in tumor-infiltrating lymphocytes and cytotoxic T cells, correlating with poorer clinical outcomes in immunotherapy [93]. Consistent with this observation, another study found that *KEAP1* mutations result in decreased dendritic cell populations and impaired T cell immune responses, underscoring the role of *KEAP1* mutations in immune escape and resistance in cancer [19]. These findings may imply that lung cancer patients with *KEAP1* mutations may respond favorably to immunotherapy. However, caution is warranted when considering immunotherapy for these patients. A study has demonstrated that *KEAP1* mutations result in the suppression of immune cells within the tumor microenvironment [93]. Also, another study has shown that patients with co-mutations of *KEAP1*, *STK11*, and *K-Ras* treated by immunotherapy had poorer progression-free survival than those without the mutations, likely due to altered immune responses and immune cell infiltration [94]. Therefore, the tumor microenvironment’s capacity to facilitate immune cell infiltration and activation is paramount in determining the efficacy of immunotherapy.

Neurofibromin 1 (NF1) is a GTPase-activating protein (GAP) that acts as an off-switch for Ras signaling by facilitating the conversion of Ras from its GTP-bound active state to its GDP-bound inactive state. Consequently, NF1 serves as a tumor suppressor by limiting the Ras-BRAF-MEK-MAPK growth signaling pathway [22]. Nonsense mutations in *NF1* result in truncated proteins that lack GAP activity, allowing Ras to remain in its GTP-bound active state, thereby continuously promoting cellular growth signals. Moreover, lung cancers harboring *EGFR* mutations exhibit increased resistance to tyrosine kinase inhibitors (TKIs) such as Erlotinib and Gefitinib when NF1 levels are low [95]. This observational study and other clinical cohort studies underscore the importance of considering a comprehensive list of genomic alterations when designing cancer treatment plans.

Epidermal Growth Factor Receptor (EGFR) is a transmembrane protein that, upon binding to its natural ligands such as EGF, activates its kinase domain, initiating a cascade of downstream signaling pathways involved in cell proliferation, survival, and differentiation. These pathways include the Ras-BRAF-MEK-MAPK, JAK-STAT, and PI3K-AKT-mTOR pathways [96]. Mutations in the *EGFR* tyrosine kinase domain are more frequently observed in lung cancers among never-smoker Asian women [97]. These mutations lead to a gain of function in the constitutive activation of EGFR, driving tumor growth. To this end, patients with *EGFR* mutations can be effectively treated with tyrosine kinase inhibitors (TKIs) such as Gefitinib and Osimertinib. However, nearly all patients treated with TKIs eventually develop drug resistance, often through the acquisition of additional TKI-resistant mutations such as T790M or through the activation of alternative pathways [98,99]. In a study, approximately 14.9% of advanced NSCLC patients with *EGFR* mutations progressed under TKI treatment and developed resistance by acquiring at least one additional genetic mutation in the PI3K pathway, such as *PIK3CA, PTEN*, and *AKT1* [100]. The authors tried to determine whether combination treatment with a TKI with an mTOR inhibitor (everolimus) yielded a better outcome, but it resulted in limited therapeutic outcomes, achieving only cancer stabilization [100]. Interestingly, in another study, the authors examined 67 biopsies from patients and revealed an inverse correlation between the *EGFR T790M* mutation and PD-L1 protein expression [101]. The authors concluded that patients with PD-L1 -/T790M+ had a better prognosis.

### 2.4. The Interconnections Among the Frequently Mutated Genes, Exosomes, and Circulating Tumor Cells

Exosomes are tiny membrane-bound extracellular vesicles secreted by both normal and cancerous cells. They play a pivotal role in intercellular communication and are enriched with a diverse array of biomolecules, including proteins, lipids, RNAs, and DNAs [102]. Exosomes are implicated in numerous biological processes, such as the modulation of immune responses, tissue repair, and the regulation of cellular homeostasis [103]. In the context of cancer, exosomes facilitate the horizontal transfer of oncogenic molecules, thereby altering the behavior of recipient cells and modulating the tumor microenvironment to promote tumor progression, metastasis, and the development of drug resistance [104,105]. Exosomes also play a critical role in regulating immune cell communication [106], inflammatory responses [107], and the tumor microenvironment [104,108]. Notably, exosomal PD-L1 can suppress immune cells to protect circulating tumor cells, thereby facilitating metastasis [109,110]. Additionally, exosomal Fas ligands can induce T cell apoptosis, contributing to immunosuppression [111].

Studies have shown that various exosomal DNAs from mutated genes, including *TP53*, *EGFR*, *ALK*, *K-Ras*, *BRAF*, *MET*, and *RB1*, were detected in 90% of patients’ plasma biopsies [112]. This suggests that cancer cells continuously shed mutated genes via exosomes, although the full significance of this phenomenon is not yet fully understood. Moreover, mutant TP53 enhances the secretion of miR-1246 via exosomes, influencing macrophage polarization to favor immune cell inactivation within the tumor microenvironment [113]. *TP53* mutations also increase tumor invasion by remodeling the extracellular matrix through the activity of cancer-associated fibroblasts [113]. Moreover, mutant EGFR-vIII [114] and mutant K-Ras [115] induce the transfer of these oncoproteins to other cells via exosomes. Furthermore, it has been shown that mutations in *EGFR* and *TP53* promote the shedding of the circulating lung tumor cells in patients [116,117]. In summary, these mutations suppress immune responses through exosomal mechanisms and enhance the dissemination of cancer cells by facilitating the circulation of circulating tumor cells.

## 3. Immunotherapy in Lung Cancer Treatments

Immunotherapy enhances the body’s immune response to fight against cancer cells. This review will focus on PD-1/PD-LA immune checkpoint immunotherapy. From 2013 to 2023, 938 immunotherapy clinical trials using PD-1/PD-L1 immune checkpoint inhibitors have been filed on ClinicalTrials.gov, and the outcomes from these data have shown positive curative benefits in treating cancer patients [118]. Besides these, other antibody-based immunotherapies will be discussed in the following section.

### 3.1. Immune Checkpoint Inhibitors (ICI): Monoclonal Antibodies Targeting PD-1/PD-L1

**Figure 1:**
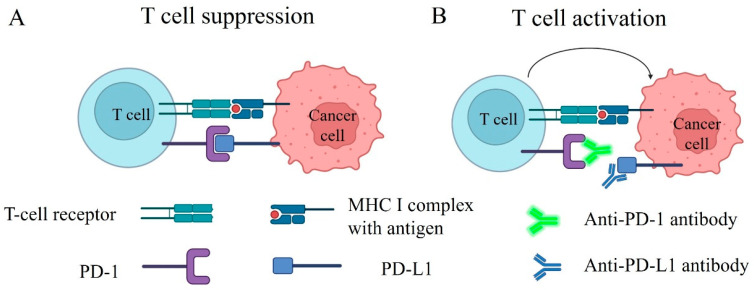
The mechanism of immunosuppression is mediated by PD-1/PD-L1 binding, and checkpoint inhibitors block PD-1/PD-L1 interaction and induce immuno-activation. The T cell receptor (TCR) identifies non-self-antigens and is activated by the antigen–MHC complex displayed on cancer cells. However, as depicted in (**A**), the T cell is suppressed when the PD-1 receptor on the T cell interacts with the PD-L1 antigen on the cancer cell. Checkpoint inhibitors can attenuate this suppression, which binds to the PD-1 receptor (represented by the blue antibody) or the PD-L1 antigen (represented by the green antibody). The binding masks the interaction sites, preventing the association between PD-1 and PD-L1. Consequently, the inhibitory effect on the T cell is lifted, allowing the T cell to initiate an attack on the cancer cell, as illustrated in (**B**).

Under normal conditions, immune cells continuously surveil the body to distinguish and eliminate non-self-entities from self by engaging the antigen–MHC complex via binding to the T cell receptor (TCR), as shown in Figure 1A. The immune system has developed checkpoint mechanisms to prevent mistakenly attacking self-entities. Immune checkpoints are immune regulatory proteins expressed on cell surfaces that maintain self-tolerance and prevent autoimmunity. PD-1 (Programmed cell Death protein 1) and CTLA-4 (Cytotoxic T-lymphocyte-associated protein 4) are the most well-studied immune checkpoints; they act as a brake on T cell’s function [119]. Besides preventing autoimmunity, checkpoint proteins also inhibit overactive immune responses; for example, antigen-presenting cells (APCs) express Programmed Death-Ligand 1 (PD-L1) and bind to PD-1 on the overactive T cell to dampen its function when the immune response needs to be downregulated, leading to inactivation of T cells, also known as T cell exhaustion [119]. Cancer cells often overexpress PD-L1, which binds to the PD-1 checkpoint proteins and inhibits T cells to escape from immune detection and destruction, as shown in Figure 1A. In recent years, immune checkpoint inhibitors (ICIs) have been developed and become the first-line immunotherapy for NSCLC [120]. ICIs are antibodies that recognize and block the interaction between checkpoint PD-1 and PD-L1 proteins to remove the inhibition of T cells. In other words, the PD-1 and PD-L1 interaction blockade activates T cells, which subsequently leads to an attack of cancer cells, as shown in Figure 1B.

Encouraging therapeutic results have been reported in using ICIs to treat lung cancers as monotherapy or adjuvant therapy with chemotherapy. For example, longer OS and PFS have been observed in metastatic NSCLC patients (without *EGFR* and *ALK* mutations) who were treated with Pembrolizumab, an ICI, in combination with chemotherapy compared to chemotherapy alone (Pemetrexed and a platinum-based chemo-drug) in a Phase 3 KEYNOTE-189 Clinical Trial (NCT02578680) [121]. With a median follow-up of 10.5 months of 616 participants, the study demonstrated that the median PFS for patients treated with the combination of Pembrolizumab and chemotherapy was 8.8 months (95% CI: 7.6–9.2). In contrast, the median PFS for patients receiving a combination of a placebo and chemotherapy was 4.9 months (95% CI: 4.7–5.2). This combination therapy significantly improved the OS in patients. Furthermore, two cases of complete remission using Pembrolizumab as monotherapy to treat advanced lung adenocarcinoma of high PD-L1 expression have been reported [122]. Moreover, the PEARLS/KEYNOTE-091 randomized triple-blinded Phase 3 Clinical Trial (NCT02504372) also showed that Pembrolizumab, as an adjuvant therapy, increased disease-free survival (DFS) in stage 1B to IIIA patients compared to placebo [123]. In this study, 1955 participants who had undergone complete surgical resection of their NSCLC (stage IB, II, or IIIA) were recruited from 196 medical centers across 29 countries. They were treated with either Pembrolizumab or a placebo over a median follow-up period of 35.6 months. The results indicated that the median DFS for patients treated with Pembrolizumab was 53.6 months (95% CI: 39.2 to not reached), compared to 42.0 months (95% CI: 31.3 to not reached) for those treated with a placebo. These findings suggest that Pembrolizumab significantly increases DFS compared to placebo. Interestingly, the authors observed that Pembrolizumab benefited patients regardless of PD-L1 expression on tumors [123]. The results suggest that PD-L1 expression level on cancer cells may not be an obligate marker for the use of ICIs, and low PD-L1 level in tumors may not be an accurate prediction marker for the poor efficacy of Pembrolizumab or possibly other ICIs. A confirmation of this claim warrants further investigation. Furthermore, other ICIs, such as Nivolumab [124,125], Atezolizumab [126,127], Durvalumab [128], and Cemiplimab, have shown robust beneficial effects on patients in different clinical trial studies, which convinced the FDA to approve these agents for treating lung cancer. These ICIs have been extensively described in the literature [118,129].

### 3.2. Prediction Markers for ICI’s Efficacy

Numerous factors influence the efficacy of immunotherapy. The following are key considerations to achieve optimal outcomes with this treatment.

#### 3.2.1. High Expression of PD-L1 in Cancer

One of the most widely studied biomarkers is the presence of PD-L1 in tumor cells. Higher levels of PD-L1 expression are often associated with better responses to PD-1/PD-L1 inhibitors [122,130]. Since cancer cells with high PD-L1 depend on it to achieve immune escape, blocking PD-L1 or PD-1 is likely to disrupt this immune escape mechanism. In addition to the high expression level of PD-L1 increasing the immune response, the ability of the tumor microenvironment to support immune cell infiltration and activation is also crucial for determining the effectiveness of immunotherapy.

#### 3.2.2. Tumor Mutational Burden (TMB)

Tumor samples with all non-silent mutations equal to 10 mutations/Mb or more are regarded as having high TMB [131]. Tumors with a high TMB are more likely to produce neo-antigens that the immune system can recognize, potentially leading to a more favorable clinical response to ICIs [132,133,134].

#### 3.2.3. DNA Repair Impairment and Microsatellite Instability

Similar to TMB, tumors with impairments in DNA repair mechanisms, such as ATM mutations [135], mismatch repair deficiency (MRD) [136], and microsatellite instability [137], are more likely to generate mutant proteins and neo-antigens, thereby eliciting a more robust immune response by ICIs.

#### 3.2.4. Mutation Profile

In addition to TMB, specific gene mutations directly influence the efficacy of cancer treatment, such as affecting the expression of the checkpoint proteins, tumor microenvironment, immune responses, etc. Table 2 below summarizes various lung cancer’s top mutated genes that have demonstrated positive and negative impacts on immunotherapy. Furthermore, a study has developed a predictive model for the efficacy of ICIs based on the mutation status of the 20 selected genes in their model. The presence of three or more mutations among these 20 genes is indicative of positive clinical outcomes from immunotherapy in the treatment of advanced non-small cell lung cancer (NSCLC) [138].

##### Favorable Mutations Leading to Positive ICI Responses

High PD-L1 expression in tumors serves as a positive predictive marker for the efficacy of ICI therapy. Studies have found that gene mutations, such as *TP53* [139], *BRAF* [139], *K-Ras* [140], *MET* [141], *EGFR-T790M* [101], and *NRF2* [142], are associated with elevated PD-L1 expression in tumors. This suggests that cancer patients harboring these mutations may be more likely to benefit from ICI therapy. Among these mutations, *TP53, MET*, and *NRF2* have also been linked to TMB, favoring better immune responses in ICI therapy.

ATM and TP53 are crucial regulators of DNA damage responses (DDRs). Mutations in these genes that impair DDRs are positively correlated with TMB, which is directly associated with the generation of neoantigens that can more effectively trigger immune responses in ICI therapy [135,143,144]. While the precise regulatory mechanism between TP53 and PD-L1 remains unclear, studies have shown that missense mutations in *TP53* lead to increased PD-L1 and IFN-γ expressions in cancer cells compared to wild-type or truncated *TP53*. This upregulation is associated with greater clinical benefits in patients undergoing ICI therapy [140]. However, the authors did not further analyze which specific *TP53* mutations contributed to the positive clinical outcomes. It is likely that different missense mutations result in varying clinical outcomes. For instance, a study demonstrated that the *TP53-R175H* mutation had a poorer clinical outcome in ICI therapy [143]. The authors observed fewer cytotoxic CD8^+^ T cells expressing PD-1 in tumors with the *TP53-R175H* mutation than those with other *TP53* missense mutations or wild-type *TP53*, suggesting that the *TP53-R175H* mutation alters the tumor microenvironment, not supporting the immune evasion of cancer cells. Further RNA sequencing analysis of tumors with the *TP53-R175H* mutation revealed significant suppression in immune response pathways, which explains the poorer clinical outcomes in patients with this mutation undergoing ICI therapy [70].

*TP53* truncated mutations did not show positive benefits; instead, they are linked to poorer clinical outcomes when treated with ICI [69]. The authors did not recommend ICI treatment for these patients. The authors further explained that more pro-tumor immune cells, such as tumor-associated M2 macrophages and neutrophils, were enriched in the tumors with *TP53* nonsense mutations than those with missense mutations. The enrichment of these pro-tumor immune cells in tumors has been linked to poorer clinical outcomes in ICI treatments [145,146].

Using a single gene mutation, such as *TP53*, as a sole predictive marker for the outcome of ICI therapy is challenging. This is because *TP53* mutations enable tumors to acquire additional genetic mutations, thereby linking *TP53* mutation to a higher TMB. Consequently, tumors with *TP53* mutations often harbor numerous other genetic alterations, each of which may positively or negatively impact the efficacy of ICI therapy. Therefore, it is recommended to obtain a comprehensive analysis of the overall genetic mutation profile rather than focusing solely on a single gene mutation.

##### Unfavorable Mutations Leading to Negative ICI Responses

ICI responses are dependent on the expression level of PD-L1 in tumors. Consequently, low PD-L1 expression is anticipated to result in poor responses to ICI therapy. It has been observed that tumors harboring mutations in *ERBB2* [139], *PIK3CA* [139], *STK11/LKB1* with *K-Ras* co-mutations [147], and *EGFR* (excluding the *EGFR-T790M* mutation) [148] exhibit low PD-L1 expression. This suggests that these tumors are more aggressive, and their growth is less reliant on immune escape mechanisms, making them less likely to respond favorably to ICI therapy.

Certain mutations have been associated with an immunosuppressive tumor microenvironment, thereby reducing the efficacy of ICIs. For instance, mutations in *ALK* [149], *KEAP1* [139], and co-mutations involving *K-Ras+KEAP1+STK11* [94] have been linked to lower levels of tumor-infiltrating lymphocytes (TILs) and CD8+ cytotoxic T cells within tumors. This reduced immune cell infiltration is positively correlated with poorer clinical outcomes.

The efficacy of ICIs is primarily predicted by two crucial factors: (1) the expression level of PD-L1 in tumors and (2) the number of activated cytotoxic T cells infiltrating the tumors, which is influenced by the tumor microenvironment. The mutual presence of high PD-L1 levels and an immune-activating microenvironment allowing T cell infiltration enhances ICI efficacy, whereas the absence of either factor diminishes therapeutic outcomes. Given the significant role of PD-L1 expression, it is worth investigating whether exogenous expression of PD-L1 in tumors, such as through the use of viral vectors or nanocarriers to deliver PD-L1 to tumor cells, can improve ICI therapy or it will cause detrimental consequences. Additionally, exploring reagents that promote tumor infiltration by immune cells may offer potential benefits as adjuvant therapies in ICI treatment.

**Table 2:** This table summarizes the most frequently mutated genes in lung cancer that positively and negatively impact the efficacy of immunotherapy.

Mutations	Reference
Resulting in positive ICI responses	
*ATM*	[135]
BRAF	[139]
EGFR-T790M mutation	[101]
*K-Ras*	[139,140,150]
*MET*	[141]
*NRF2*	[142]
*TP53* missense mutations, but not nonsense mutations (missense mutation point unspecified in this study)	[143]
*TP53+TTN* co-mutations	[144]
Resulting in negative ICI responses	
*ALK*	[149]
*ERBB2*	[139]
*EGFR* with low PD-L1 expression	[148]
*K-Ras+KEAP1+STK11* co-mutations	[94,151]
*KEAP1*	[139]
*PIK3CA*	[139]
*STK11/LKB1+K-Ras* co-mutations	[147]
*TP53* (truncation)	[69]
*TP53-R175H* missense mutation	[70]

### 3.3. Immunomodulating Effects of Natural Products on Immunotherapy

Research has demonstrated that natural products may enhance the therapeutic efficacy of immunotherapy. For instance, quercetin has been extensively studied for its direct anticancer effects [152], including down-regulating the EGFR-T790M mutant protein level [153], inhibiting EGFR mutant signaling [154], directly inhibiting EGFR’s kinase activity [155], inducing apoptotic pathways [156,157], and inhibiting survival and proliferation pathways [157,158,159], as evidenced in lung and other cancer cells [160,161]. Additionally, quercetin has been shown to augment the effects of immunotherapy in animal models by increasing CD8+ T cell infiltration in tumors and blocking the interaction between PD-L1 and PD-1 [162]. Furthermore, quercetin mitigates tumor-associated inflammation, known to facilitate tumor development, progression, and metastasis [152,163,164]. A study highlighted that the combination of quercetin and a PD-L1 checkpoint inhibitor improved the tumor microenvironment in a liver cancer model, suggesting quercetin’s potential as an immunotherapy adjuvant in addition to its anti-cancer potential [165].

Other natural products have also demonstrated potential as immunotherapy adjuvants. For instance, triterpenoid saponin has been shown to inhibit the interaction between PD-1 and PD-L1 through the down-regulation of STAT3 [166]. Additionally, the ethanolic extract of Mahonia aquifolium flowers [167], various mushroom extracts [168,169], ginsenoside of Panax Ginseng [170], Korean Red Ginseng [171], and Astragalus membranaceus [172] exhibit immunomodulatory effects. The utilization of natural products in modern medicine remains a largely underexplored area. Therefore, further research in this field is essential.

## 4. Monoclonal Antibodies Targeting Cell Surface Receptors and Antibody–Drug Conjugates (ADCs)

According to the TCGA database, 9% of lung cancer patients harbor *EGFR* mutations, while 2% exhibit *ERBB2* mutations. Another study reported higher frequencies, with *EGFR* and *ERBB2* mutations present in 28% and 6% of cases, respectively [173]. Notably, up to 90% of East Asian women with lung cancer who have never smoked were found to have *EGFR* mutations in their tumors [174]. These activating mutations lead to increased expression levels of these cell surface receptors. Additionally, surface antigens such as mesothelin [175], Notch2/3 [176], and TROP2 [177] are up-regulated in lung cancer. Consequently, various monoclonal antibodies have been developed to target these receptors for therapeutic purposes. Compared to tyrosine kinase inhibitors (TKIs), monoclonal antibodies are less commonly used in lung cancer treatment and are mostly in the investigational stage [178]. However, they hold significant potential and are expected to play an important role in the future of lung cancer therapy.

**Figure 2:**
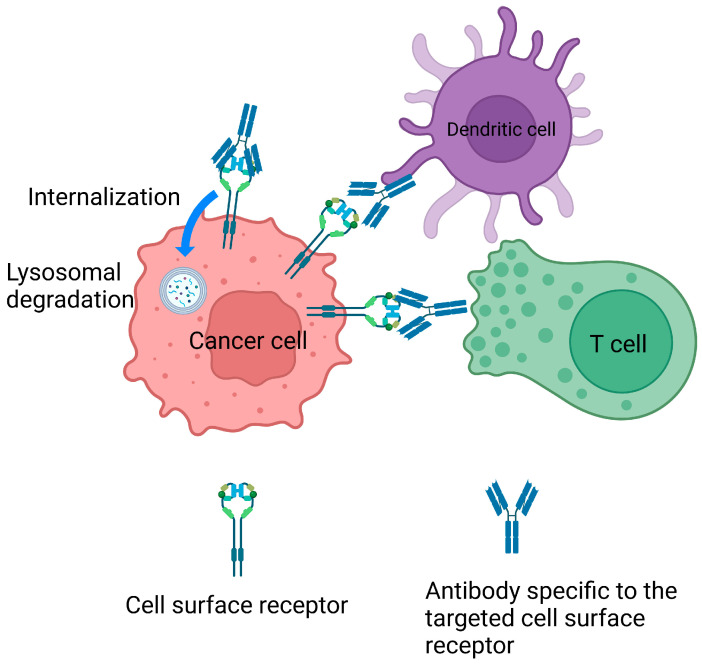
Mechanism of monoclonal antibody targeting in cancer therapy. The monoclonal antibody binds to the extracellular domain of cancer cell surface receptors, forming a complex that is subsequently internalized into the lysosome for degradation. Additionally, the antibody–receptor complex activates an immune response involving T cells and dendritic cells.

Necitumumab serves as an example to illustrate the pharmacodynamic properties of monoclonal antibodies. Necitumumab is a recombinant immunoglobulin G1 antibody that targets the extracellular domain of the EGFR receptor. Upon binding to EGFR surface receptors, the antibody–receptor complex triggers both innate and adaptive immune responses, as depicted in Figure 2. For instance, increased infiltration of immune cells such as T cells, dendritic cells, and NK (not shown in the figure) cells has been observed in head and neck cancer biopsies following anti-EGFR monoclonal antibody treatment [179]. Furthermore, anti-EGFR monoclonal antibody induces the internalization of EGFR into lysosomes, leading to subsequent lysosomal degradation of the receptors, as observed in colon cancer cells [180,181]. This process often results in the down-regulation of surface receptor expression. Table 3 lists the FDA-approved monoclonal antibodies that can be used to treat lung cancer, along with their mechanisms and molecular targets.

Monoclonal antibody-targeted therapy can be used as a monotherapy or in combination with chemotherapy. For example, the use of Necitumumab plus chemotherapy (gemcitabine and cisplatin) has shown improved OS in NSCLC patients [182]. Other studies also observed a beneficial OS and response rate when a combination of Cetuximab plus chemotherapy was given to NSCLC patients [183,184]. However, a research group vowed that Cetuximab has limited benefit to NSCLC patients, requesting more accurate prediction markers for Cetuximab usage before monoclonal antibody therapy becomes routine in clinical settings [178].

Bevacizumab and Ramucirumab are monoclonal antibodies that target and inhibit the angiogenesis pathway, specifically the formation of new blood vessels [185,186]. Recognizing that anti-angiogenesis alone is often insufficient to fully inhibit cancer growth, these monoclonal antibodies are typically administered in combination with other chemotherapy or immunotherapy agents to enhance clinical benefits [187,188].

In addition to the use of monoclonal antibodies for targeting cancer cells, ADCs have been developed. ADCs represent a class of targeted cancer therapies that combine specific monoclonal antibodies with chemotherapy drugs, thereby enhancing the precision and efficacy of cancer treatment [189]. For example, Trastuzumab deruxtecan is an anti-ERBB2 antibody conjugated with topoisomerase inhibitor for NSCLC with *HER2* mutation [190] and an anti-CD20 antibody conjugated with a nanoparticle loaded with paclitaxel for B-cell lymphoma [191]. A few ADCs for lung cancer treatment have been listed in Table 3.

**Figure 3:**
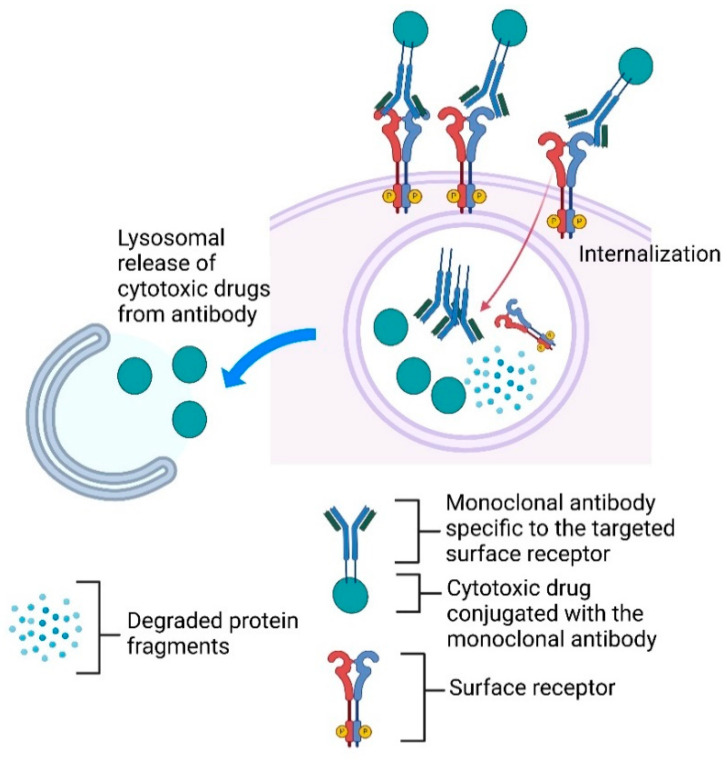
**Schematic of an antibody–drug conjugate (ADC).** The ADC is composed of a monoclonal antibody linked to a cytotoxic drug. The monoclonal antibody specifically targets a cell surface receptor. Upon binding to the receptor, the antibody–receptor complex is internalized into the cell and directed to lysosomes. The complex undergoes degradation within the lysosome, releasing the cytotoxic drug, which subsequently kills cancer.

ADCs selectively deliver cytotoxic drugs directly into cancer cells by targeting specific cell surface receptors, thereby minimizing damage to normal cells. Upon binding to the target receptors, the ADC–receptor complex is internalized and transported to the lysosome for degradation. The acidic environment within the lysosomal compartment facilitates the release of the cytotoxic drug from the antibody. Subsequently, the released cytotoxic drugs enter the cytoplasm and induce cell death, as illustrated in Figure 3. Thus, ADCs down-regulate cancer cell surface receptors, thereby dampening growth signals and effectively delivering cytotoxic agents to eradicate cancer cells. Furthermore, studies have shown that cytotoxic drugs from ADCs are released to kill neighboring cancer cells, maximizing cancer elimination [192]. Moreover, ADCs have demonstrated the ability to resensitize chemo-resistant cancer cells, rendering them chemo-sensitive. For instance, an ADC conjugated with VERU-111, a novel tubulin inhibitor, has been shown to resensitize paclitaxel-resistant cancer cells, thereby enhancing their susceptibility to treatment and improving cancer cell eradication [193].

**Table 3:** Summarizes a list of FDA-approved monoclonal antibodies used to treat lung cancers.

Monoclonal Antibody Target	Drug	Mechanism(s)	References
Mesothelin	Amatuximab	Inhibition of mesothelin growth signal	[194]
EGFR	Cetuximab	Inhibition of EGFR growth signal	[195]
	Necitumumab		[196]
	Panitumumab		[197]
ERBB2	Trastuzumab	Inhibition of ERBB2 growth signal	[195]
	Trastuzumab deruxtecan (ADC)	Inhibition of ERBB2 growth signal and inhibition of DNA replication	[190]
	Trastuzumab emtansine (ADC)	Inhibition of EGFR growth signal and internationalization and release of the microtubule inhibitor DM1	[198]
Notch 2/3	Tarextumab	Inhibition of growth signal	[199]
TROP-2	Sacituzumab Govitecan (ADC)	Induction of TROP-2 mediated internationalization and release of the topoisomerase inhibitor SN-38	[200,201]
VEGF-A	Bevacizumab	Inhibition of angiogenesis	[185]
VEGFR2	Ramucirumab		[186]

### Factors Affecting Monoclonal Antibodies Targeting Cell Surface Receptors and ADCs

Several factors influencing the efficacy of monoclonal antibodies targeting cell surface receptors and ADCs are outlined in Table 4. For example, the administration of these therapies involves foreign antibodies, which may trigger an immune response, leading to a shorter half-life and reduced drug effectiveness [202]. Additionally, issues such as the failure of monoclonal antibodies to internalize [203] and impaired antibody-mediated cellular cytotoxicity [204] have been observed, both of which diminish the effectiveness of cancer elimination. Moreover, the drug component of ADCs is intended to be released within the tumor’s lysosome. However, if the drug is unstable and released into the patient’s systemic circulation, it can cause adverse side effects [205]. Conversely, defective lysosomal function can hinder the release of conjugated cytotoxic drugs, thereby reducing drug availability within the tumor [203]. Given that only a small fraction of cytotoxic drugs may be released within cancer cells, the efficiency of drug payload release is critical to therapeutic efficacy [206]. Additionally, the development of drug resistance mechanisms, such as the expression of the P-glycoprotein (Pgp) drug efflux transporter on the cell surface, can further reduce drug availability in cancer cells [203].

## 5. Bispecific Antibodies

**Figure 4:**
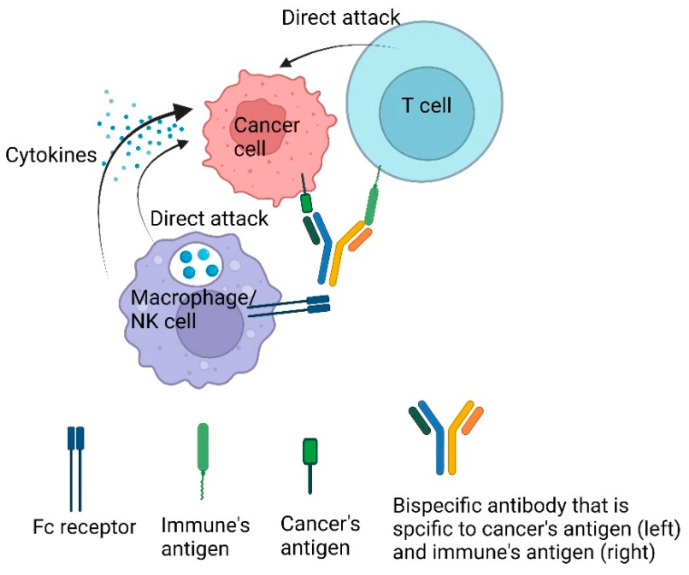
**Working principle of a bispecific antibody.** Bispecific antibodies are engineered in the form of Immunoglobulin G (IgG) through genetic engineering. One-half of the IgG is designed to recognize the T cell’s antigen on cytotoxic T cells, while the other half is tailored to identify a specific antigen in cancer cells. This specificity allows the bispecific antibody to target any antigen of interest specific to cancer antigen in a specific patient. The function of the bispecific antibody is to bridge the T cell and cancer cell, facilitating close proximity for T cell-mediated cancer destruction. Additionally, the innate immune system, such as macrophages or NK cells, uses the Fc receptor to recognize the bispecific antibody and subsequently releases cytokines to enhance cancer elimination or by direct cancer attack.

Bispecific antibodies are immunoglobulin G (IgG) engineered to contain two different Fab (fragment antigen binding) regions. One Fab region recognizes the cancer’s specific antigen, while the other Fab region recognizes the immune cell’s antigen. Thus, this bispecific-targeting capability allows them to bring cancer cells and immune cells into close proximity, thereby enhancing the immune system’s ability to recognize and destroy cancer cells (Figure 4). By engaging both the tumor and immune cells, bispecific antibodies can effectively disrupt cancer cell signaling pathways and promote a more robust anti-tumor response, for example, by engaging innate macrophages and natural killer cells [207]. Different subtypes of bispecific antibodies have been developed under the same working principle, which has been described in the literature in detail [208].

To date, the FDA has approved two bispecific antibodies for lung cancer treatment, Tarlatamab and Amivantamab. Tarlatamab is indicated for late-stage SCLC patients, who otherwise have a five-year survival rate of only 3–7% without such treatment [209]. The second approved bispecific antibody is Amivantamab, which is used for NSCLC with *EGFR* exon 20 insertion mutations [210]. While more than eight bispecific antibodies have received FDA approval for treating various types of cancer; this review will not cover those agents.

Tarlatamab targets DDL3, a surface antigen aberrantly expressed in SCLC cells, and simultaneously recognizes CD3 antigen on T cells to allow direct contact with T cells, thereby eliciting a potent immune response [209]. Amivantamab is a bispecific antibody designed to simultaneously recognize and bind to two cell surface receptors on cancer cells: EGFR and MET. While it does not target T cell receptors, the combination treatment of Amivantamab with chemotherapy (carboplatin and pemetrexed) has demonstrated superior clinical outcomes compared to chemotherapy alone [211].

Since both checkpoint inhibitors and bispecific antibodies rely on tumor infiltration and T cell activation, the predictive biomarkers for their efficacy are likely to be similar. For instance, gene mutations that alter the tumor microenvironment and suppress lymphocyte activation negatively impact the efficacy of bispecific antibodies. Additionally, high expression of PD-L1 on tumors serves as a negative marker for bispecific antibodies because, despite the proximity of T cells to cancer cells, the interaction between PD-1 and PD-L1 prevents T cell activation. Therefore, bispecific antibodies should be considered for use in patients with low PD-L1 expression.

## 6. Further Perspectives on Lung Cancer Therapy

Besides the current lung cancer therapeutic approaches, numerous new technologies have emerged, including gene therapy, cancer vaccines, Antibody-based Proteolysis-Targeting Chimeras (PROTACs), etc. Given that lung cancer is predominantly driven by gene mutations, as listed in Table 1, there is potential to cure cancer by correcting these genetic abnormalities. Currently, gene editing technology, such as CRISPR-Cas9, is advancing rapidly, enabling the precise excision of mutated genes and their replacement with correct DNA fragments to achieve gene therapy [212]. One crucial concern about gene therapy is the limitation of gene delivery into the tumor cells, and this limitation can be solved by inhalable gene transfection technology [213] as well as delivery by nanoparticles [214]. Thus, gene therapy holds significant potential as a future treatment for lung cancer.

Secondly, similar to immunotherapy, cancer treatment can leverage immune cells to target and eliminate cancer cells by recognizing specific cancer antigens. By identifying cancer-specific cell surface markers, we can synthesize these antigens and immunize the human body to mount an immune response against cells expressing these markers, thereby creating a cancer vaccine. Recently, BioNTech, a pharmaceutical company specializing in mRNA vaccines, invented the first mRNA cancer vaccine, namely BNT116, and it launched the first cancer vaccine for NSCLC in 2024 over 34 research sites. If this mRNA vaccine proves successful, it has the potential to significantly revolutionize cancer treatment.

Thirdly, PROTAC is a novel technology that could specifically target any protein of interest, such as cancer cell surface markers (such as EGFR, ERBB2, and PD-L1), and induce its ubiquitination proteasome degradation [215,216,217,218]. This approach can facilitate the down-regulation of activated receptors and PD-L1 proteins in lung cancer, thereby decreasing the growth-promoting signal [219] and enhancing the immune response [220]. These advancements represent promising possibilities in the realm of cancer therapy.

## 7. Conclusions

Decades of cancer research have yielded extensive knowledge about the genetic mutations associated with lung cancer. This has led to the development of small molecule therapies specifically designed to target these mutations and eradicate cancer cells. However, these treatments often exert selective pressure, allowing the emergence and proliferation of cancer cells that acquire additional mutations to circumvent drug inhibition. Immunotherapy offers a potential solution to drug resistance by enhancing the ability of immune cells to target and kill cancer cells. Various immunotherapeutic approaches, including immune checkpoint inhibitors, monoclonal antibodies, antibody–drug conjugates, and bispecific antibodies, have demonstrated promising therapeutic effects. Future research should focus on selecting patients for immunotherapy based on their specific genetic mutations, which could serve as predictive markers for the efficacy of immunotherapeutic interventions. Other novel approaches, such as gene editing, cancer vaccine, PROTAC, and natural products can potentially be applied to lung cancer therapy in the future.

## Figures and Tables

**Table 1:** The top commonly mutated genes in lung cancers based on the database from the TCGA Cancer Genome Atlas Program of three combined cohort studies (TCGA-lung squamous cell carcinoma (LUSC) [10], TCGA-lung adenocarcinoma (LUAD) [5], and CDDP-EAGLE-1) consist of a total of 1099 lung cancer samples.

Gene	Mutation Rate (%)	Top 3 Hotspot Missense or Nonsense Mutations on Protein	Consequences	Ref.
*TP53*	65	R175H, R248Q, R273C	Loss of tumor suppressor functions	[11]
*KRAS*	16	G12C, G12V, G12D	Gain of growth signals and immune escape	[12,13,14,15]
*KEAP1*	14	R470C, V271L, G480W	Decrease in death signals and immune responses	[16,17,18,19]
*NF1*	11	R2450 *, R1362 *, R440 *	Loss of tumor suppressor functions	[20,21,22,23]
*EGFR*	9	L858R, G598V, A289V	Gain of growth signals	[14,24,25]
*NRF2*	8	R34G, D29H, E79Q	Gain of growth signals	[17,26,27]
*ATM*	8	L2307F, R337C, P604S	Loss of DNA repair functions	[28]
*ALK*	6	R1275Q, F1174L, E296K	Gain of growth signals	[29,30,31]
*Rb1*	6	R552 *, R320 *, R556 *	Loss of growth inhibition functions	[32]
*BRAF*	5	V640E, V640M, D634N	Gain of growth signals	[14,33]
*MET*	3	M1268T, S213L, R1166Q	Gain of growth signals	[14,34]
*ERBB2*	2	S310F, V842I, L755S	Gain of growth signals	[35,36,37]

* Nonsense mutation.

**Table 4:** Factors affecting the efficacy of monoclonal antibodies targeting cell surface receptors and antibody–drug conjugates (ADCs).

Factors Affecting the Efficacy of Monoclonal Antibodies Targeting Cell Surface Receptors and ADCs	Reference
1. The pharmacokinetic and immunogenicity of the antibody	[202]
2. Failure of internalization of the antibody	[203]
3. Impaired antibody-mediated cellular cytotoxicity	[204]
**Factors affecting the efficacy of ADCs**	
1. Stability of cytotoxic drug conjugate in systemic circulation	[205]
2. Cytotoxic drug efflux mechanism in cancer	[203]
3. Defective lysosome function	[203]
4. Tumor-specific payload	[206]

## Abbreviations

ADC	Antibody–drug conjugate
ALK	Anaplastic lymphoma kinase
APCs	Antigen-presenting cells
ATM	Ataxia telangiectasia mutated
BRAF	v-raf murine sarcoma viral oncogene homolog B
CFDA	China Food and Drug Administration
CTLA-4	Cytotoxic T-Lymphocyte-Associated protein 4
EGFR	Epidermal growth factor receptor
ERBB2	v-erb-b2 avian erythroblastic leukemia viral oncogene homolog 2
ICIs	immune checkpoint inhibitors
KEAP1	Kelch-like ECH-associated protein 1
KRAS	Kirsten rat sarcoma virus
MEK	MAPK/ERK kinase
MET	Hepatocyte Growth Factor Receptor
MRD	Mismatch repair deficiency
NF1	Neurofibromatosis type 1
NRF2	NFE2 Like BZIP Transcription Factor 2, also known as NEF2L2
NSCLC	Non-small-cell lung cancer
OS	Overall survival
PD-1	Programmed cell Death protein 1
PD-L1	Programmed Death-Ligand 1
PROTACs	Antibody-based Proteolysis-Targeting Chimeras
Rb1	Retinoblastoma Transcriptional Corepressor 1
SCLC	Small-cell lung cancer
TKI	Tyrosine kinase inhibitor
TMB	tumor mutational burden
TP53	Tumor suppressor protein p53
TTN	Titin
U.S. FDA	U.S. Food and Drug Administration
